# Supplementary material for: Genistein Activates Transcription Factor EB and Corrects Niemann–Pick C Phenotype
Source: Int J Mol Sci. 2021 Apr 19;22(8):4220. doi: 10.3390/ijms22084220 (PMC8073251; doi:10.3390/ijms22084220)
Supplement: Supplementary file 1 [file ijms-22-04220-s001.zip › ijms-1123227-supplementary.pdf]

Genistein Activates Transcription Factor EB and Corrects Niemann–Pick C Phenotype

Graciela Argüello [1,2,*], Elisa Balboa [1], Pablo J. Tapia [1,3], Juan Castro [1], María José Yañez [1], Pamela Mattar [4],
Rodrigo Pulgar [5] and Silvana Zanlungo [1,*]

1 Departament of Gastroenterology, School of Medicine, Pontificia Universidad Católica de Chile, Santiago 3580000, Chile; ebalboa@gmail.com (E.B.); pablo.tapia.o@gmail.com (P.J.T.); jcastros@puc.cl (J.C.); myanezh@gmail.com (M.J.Y.)
2 School of Health Sciences, Universidad Católica del Maule, Talca 3466706, Chile
3 Center for Cell Biology and Biomedicine (CEBICEM), School of Science and Medicine, Universidad San Sebastián, Santiago 7510235, Chile
4 Departament of Physiology, School of Biological Science, Pontificia Universidad Católica de Chile, Santiago 3580000, Chile; pamela.mattararanguiz@einsteinmed.org
5 Laboratory of Genomics and Genetics of Biological Interactions, Instituto de Nutrición y Tecnología de los Alimentos (INTA), Universidad de Chile, Santiago 7830490, Chile; rpulgar@inta.uchile.cl
* Correspondence: graciela.arguelloflorencio@gmail.com (G.A.); szanlungo@uc.cl (S.Z.); Tel.: +56-2-23543820 (S.Z.)

Supplementary Table S1**.**Primer sequences (from 5′ to 3′)

TFEB: Forward GCGGCAGAAGAAAGACAATC. R**everse** CTGCATCCTCCGGATGTAAT

CTSB: Forward TTAGCGCTCTCACTTCCACTACC. R**everse** TGCTTGCTACCTTCCTCTGGTTA

CTSD: Forward CTTCGACAACCTGATGCAGC. **Reverse** TACTTGGAGTCTGTGCCACC

LAMP1: Forward CCAACTTCTCTGCTGCCTTC. **Reverse** AGCAATCACGAGACTGGGG

ATP6V1HRT: Forward GGAAGTGTCAGATGATCCCCA. **Reverse** CCGTTTGCCTCGTGGATAAT

TPP1: Forward GATCCCAGCTCTCCTCAATACG. **Reverse** GCCATTTTTGCACCGTGTG

ATG9B: Forward TTCCTTTGCCCTTATGGATG. **Reverse** AACCGCATCAAAGAAAGCTC

BECN1: Forward GGTGTCTCTCGCAGATTCATC. **Reverse** TCAGTCTTCGGCTGAGGTTCT

MCOLN1: Forward GAGTCCCTGCGACAAGTTTC. **Reverse** TGTTCTCTTCCCGGAATGTC

VPS11: Forward CAAGCCTACAAACTACGGGTG. **Reverse** GAGTGCAGAGTGGATTGCCA

WlPl1: Forward GCCTATCCTGGAAGCCTGAC. **Reverse** TGATGGCAGCTAGTGTTCCC

GAPDH: Forward ATGTTCGTCATGGGTGTGAA. **Reverse** AGGGGTGCTAAGCAGTTGGT
